# Cardiac Papillary Fibroelastoma: Atypical Presentation Mimicking Infective Endocarditis With False Positive Commensal Blood Cultures

**DOI:** 10.7759/cureus.42660

**Published:** 2023-07-29

**Authors:** Carlos J Collado-Rivera, Krisela Vojniku, Mohit Sharma, Harold A Fernandez, Alan T Kaell

**Affiliations:** 1 Internal Medicine, Mather Hospital Northwell Health, Port Jefferson, USA; 2 Cardiology, Mather Hospital Northwell Health, Port Jefferson, USA; 3 Cardiothoracic Surgery, South Shore University Hospital Northwell Health, Bay Shore, USA

**Keywords:** case reports, primary cardiac tumors, infective endocarditis, valvular lesions, cardiac tumors, cardiac papillary fibroelastoma, benign cardiac tumor

## Abstract

Cardiac papillary fibroelastomas (CPFs) are rare benign cardiac neoplasms that carry a high risk of embolization if not diagnosed and managed in a timely manner. As most patients are asymptomatic, CPF may be incidentally detected on transthoracic echocardiography (TTE) when performed for other indications. Management of incidental CPF in asymptomatic patients is debatable. We report an unusual case of an incidental CPF in an asymptomatic patient admitted to the hospital for presumed infective endocarditis (IE). Two weeks following laser resection of laryngeal cancer (LC), a new pansystolic murmur was audible during a routine cardiology visit. Outpatient TTE revealed a “vegetation-like” lesion on the mitral valve (MV). Blood cultures (BC) with Gram-positive cocci in clusters (GPC) were reported within 24 hours. This prompted hospital admission for empiric antibiotics. A transesophageal echocardiogram (TEE) confirmed the lesion to be an echogenic mass attached to the MV consistent with CPF. Repeat BC, prior to empiric antibiotic initiation, were all negative. In the absence of all other signs and symptoms of IE, it was determined that the initial BC was false positive and IE was ruled out. Surgical resection was performed due to the potential risk of embolization. The pathology confirmed the diagnosis of CPF with negative tissue cultures.

## Introduction

Primary cardiac tumors are extremely rare, representing 5-10% of all tumors of the heart with autopsy incidence of 0.002-0.3% [1] and prevalence of 0.001-0.03% [2]. Nearly 75% are benign, while 25% are malignant. On the contrary, secondary cardiac tumors (metastatic) are far more frequent, approximately 30- to 40-fold more prevalent than primary tumors [3]. Cardiac papillary fibroelastomas (CPFs) are relatively small, rare benign neoplastic primary tumors of the heart, the second most common after myxomas [4], but the most common valve tumors with a prevalence of less than 0.1% [5]. Eighty percent occur on valvular surfaces, while 20% occur on extra-valvular surfaces [6]. The distribution of valvular location is aortic valve (35-63%), mitral valve (MV) (9-55%), tricuspid valve (6-15%), and pulmonic valve (0.5-8%) [7]. In extreme cases, CPFs have been identified on non-valvular tissue such as the ascending aortic wall [6]. Typically, CPFs are asymptomatic and incidentally discovered on routine cardiac examinations, cardiac surgery, or autopsy; however, some patients may present with neurological and/or cardiac complications. Diagnosis can be challenging and management somewhat controversial. Here, we present a patient who was admitted to the hospital for presumed infective endocarditis (IE) but had a CPF. Pertinent literature on CPF mimicking IE and management decisions are discussed.

This study was previously presented as a poster at the 2023 Medical Society of the State of New York's (MSSNY) 16th Resident/Fellow/Medical Student Poster Symposium, Tarrytown, NY.

## Case presentation

A 79-year-old male, with a medical history of essential hypertension, coronary artery disease, dyslipidemia, anxiety, chronic pain, and laryngeal cancer (LC), was sent to the emergency department for presumed IE after a routine cardiology follow-up revealed a new pansystolic murmur, transthoracic echocardiography (TTE) demonstrated a “vegetation like” lesion on the MV, and positive blood cultures (BC) were reported as growing Gram-positive cocci in clusters (GPC). Importantly, two weeks prior he had undergone laser excision of his LC. Although he was completely asymptomatic, the high clinical suspicion for IE in this setting prompted hospital admission for empiric treatment with intravenous vancomycin and further evaluation.

On physical examination vital signs were as follows: he was afebrile, blood pressure (BP) 150/75 mmHg, heart rate (HR) 76 bpm, respiratory rate (RR) 16 breaths/min, and O_2_ saturation was 95% in room air. An unchanging pansystolic II/VI murmur at the cardiac apex was audible, but no focal neurological deficits or cutaneous or retinal lesions were appreciated. Chronic bilateral non-pitting lower extremity edema was noted. Laboratory results showed increased WBC with left shift, increased ESR, decreased sodium and bicarbonate, and increased glucose levels (Table 1).

**Table 1:** Pertinent abnormal laboratory results. H: high; L: low

Lab values	Results	Reference range
White blood cell count (WBC) (K/uL)	12.19 (H)	3.8-10.5
Neutrophils (%)	82.0 (H)	13-44
Lymphocytes (%)	6.6 (L)	13-44
Absolute neutrophil count (K/uL)	10.0 (H)	1.8-7.4
Lymphocytes, absolute (K/uL)	0.81 (L)	1.0-3.3
Erythrocyte sedimentation rate (ESR) (mm/h)	29 (H)	0-20
Sodium (mmol/L)	133 (L)	136-145
Bicarbonate (mmol/L)	21 (L)	22-29
Glucose (mg/dL)	116 (H)	74-109

**Figure 1:**
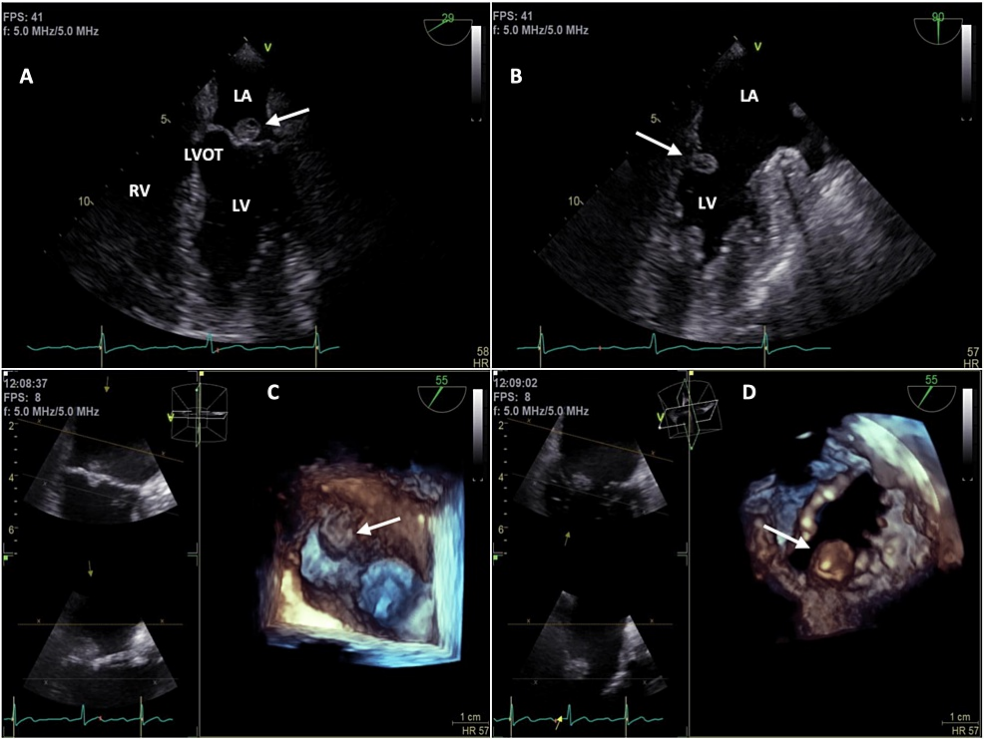
Transesophageal echocardiogram showing a 1.2×1.4 cm echogenic mass attached to the atrial side of the posterior mitral leaflet (white arrows). (A and B) Apical view during systole and diastole, respectively. (C and D) Three-dimensional view during systole and diastole, respectively. Note mass moving into the left ventricle during diastole (B and D). LA: left atrium; LV: left ventricle; LVOT: left ventricular outflow tract; RV: right ventricle

Electrocardiogram showed chronic first-degree atrioventricular block. A repeat TTE showed a definite spherical, solid vegetation on the tip of the anterior leaflet of the MV and left ventricular ejection fraction of 66%. TEE demonstrated a 1.2×1.4 cm echogenic mass attached to the posterior mitral leaflet on the atrial side that was moving into left ventricular cavity, suspicious for CPF (Figures 1A-1D). No endocardial vegetations were seen.

The positive outpatient BC identified a commensal, non-pathogenic *Staphylococcus hominis,* and repeat BC prior to the initiation of antibiotics were persistently negative for five days. Antibiotics were discontinued. Due to the potential risk of tumor embolization, surgery was proposed. Six months later, the patient was scheduled for open-heart excision of the tumor. Transesophageal echocardiogram (TEE) was used intraoperatively to guide anesthesiologists and surgical team. The live recording with TEE re-demonstrated the echogenic mass moving into the left ventricular cavity during diastole (Videos 1, 2).

**Video 1:** Intraoperative transesophageal echocardiogram showing the echogenic mass moving into the left ventricle during diastole.

**Video 2:** Three-dimensional view of intraoperative transesophageal echocardiogram showing the echogenic mass moving into the left ventricle during diastole.

**Figure 2:**
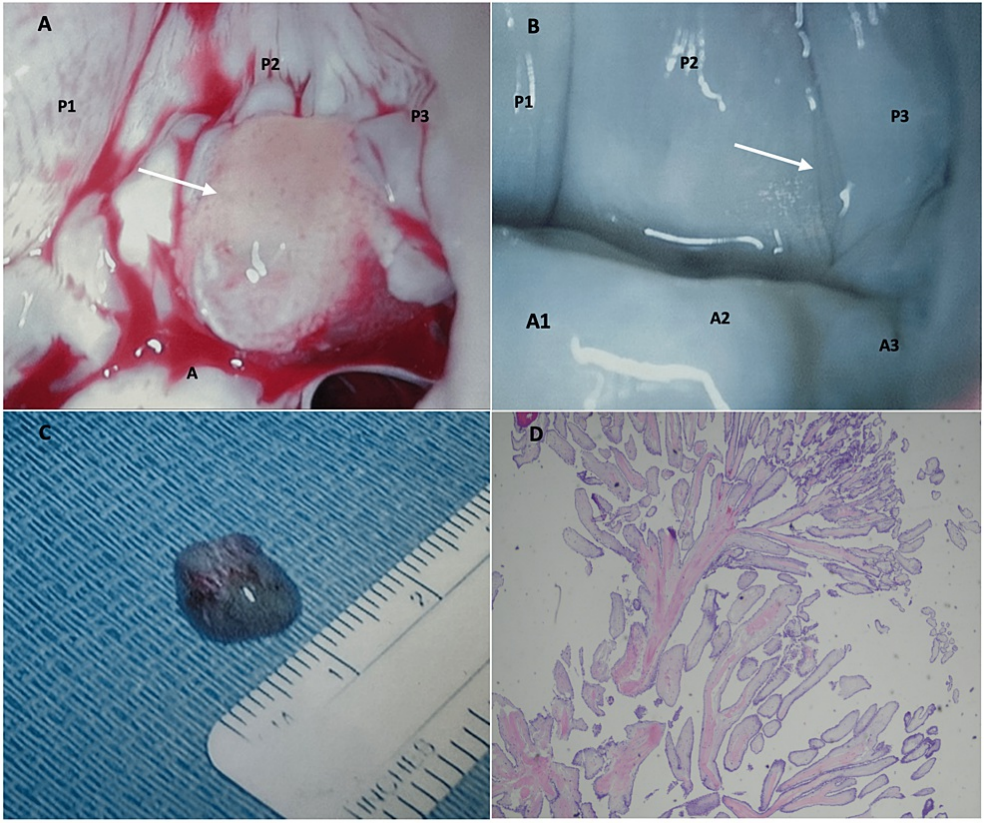
Histological examination of the intracardiac mass during and post-open-heart surgery. The images show (A) mass attached between P2 and P3 indentation of the posterior MV leaflet (black arrow), (B) MV appearance post-surgical resection with complete preservation of the posterior leaflet (white arrow), (C) 1×1×0.6 cm of tan-white entirely papillary fragment of soft tissue mass, (D) hematoxylin and eosin staining showing avascular papillary tissue with a surrounding layer of endothelium. MV: mitral valve; A1: anterior leaflet segment; A2: middle leaflet segment; A3: posterior leaflet segment; P1: anterior or medial leaflet scallop; P2: middle leaflet scallop; P3: posterior or lateral leaflet scallop

The patient underwent surgical resection of the mass with complete preservation of the MV (Figures 2A-2C). Histological examination confirmed the diagnosis of CPF (Figure 2D). The post-operative course was uneventful.

## Discussion

CPF is the most common primary benign neoplasm of the heart valves. Although extremely rare, prompt diagnosis and intervention are important to prevent cardiac and neurologic complications. In fact, the first reported case was in 1975 as an embolic complication leading to myocardial infarction [8]. Since then, CPF has been identified as potential etiology of embolic stroke, cardiac arrythmias, coronary artery embolization, and even sudden cardiac death.

Most cases are incidentally diagnosed with echocardiogram in asymptomatic patients, usually around the sixth to eighth decade of life, while others have been identified by one or a combination of the above-mentioned symptoms. However, few cases have been reported in which a diagnosis of CPF was made during patient’s evaluation for suspected IE. For this reason, we presented a case of a male in his eighth decade of life who was incidentally found to have a CPF after being admitted to the hospital for presumed IE. While the combination of the recent LC procedure, initial positive BC, new heart murmur, and TTE findings were highly suspicious for IE, TEE findings demonstrated an intracardiac tumor (Figures 1A-1D). A live recording on TEE demonstrated a mass moving into the LV cavity during diastole (Videos 1, 2).

As our patient was asymptomatic, anticoagulation was not indicated but recommended outpatient follow-up for tumor resection due to increased risk of embolization. On presurgical evaluation, the patient was also found to have severe stenosis of the proximal left anterior descending artery. Patient underwent open-heart excision of the tumor and coronary artery bypass surgery. The mass was located at the indentation between P2 and P3 leaflets scallops of the MV (Figure 2A). The MV was preserved after mass resection (Figure 2B). Macroscopically, the mass consisted of a 1×1×0.6 cm tan-white entirely papillary fragment of soft tissue (Figure 2C). Microscopically, hematoxylin and eosin (H&E) staining showed avascular papillary tissue with a surrounding layer of endothelium, confirming the diagnosis of CPF (Figure 2D).

The etiology of CPF still remains unclear. The most accepted theory is that they originate as microthrombi at valvular endocardium where endothelial damage has occurred, and with time, these evolve into excrescences and eventually CPF [9]. On echocardiogram, they range in size from 2 to 40 mm, and in-vivo can be as small as 2 mm with an average size of 9 mm. The macroscopic appearance varies in shape, commonly, consisting of frond-like arms emanating from a stalked central core, but can also be ovoid, soft, gelatinous white fragment of soft tissue as seen in our patient (Figure 2C) [10]. The histopathological findings show multiple elongated branched papillary fronds consisting of an acellular matrix surrounded by a single layer of endothelial cells (Figure 2D). 

Imaging modalities for the diagnosis include TTE, TEE, cardiac MRI, and CT. TTE has excellent diagnostic sensitivity and specificity (88.8% and 88.7%, respectively). However, when a smaller CPF (<0.2 cm) is suspected, TEE has greater sensitivity (76.6%) than TTE (61.9%) [10]. In addition, echocardiogram shows a mobile pedunculated or sessile mass on the valvular endocardium that moves during systole and diastole. A three-dimensional echocardiogram can determine the diameter of the mass, especially to aid in therapeutic decision-making, particularly surgical access prior to resection [11]. Diagnosis is confirmed by histological examination of the resected intracardiac mass.

No specific guidelines exist for treatment. Surgical excision is the recommended treatment unless there is a contraindication. In fact, a study analysis of the resection of CPF over the years 1998-2020 showed a low operative mortality (0-2.5%), low recurrence rate (15.8%), and a high estimated survival (78.4%) at 10 years [12]. The indication for operation is determined by the patient’s overall characteristics and comorbidities/risk factors, presence or absence of symptoms, the type of tumor, size, location, and operative and long-term outcomes. In the case of CPF, because of their fragile nature, they carry a high risk of embolic complications; therefore, most authors propose surgical management even in asymptomatic patients [13]. If the patient is a poor surgical candidate and yet symptomatic, anticoagulation and close monitoring are reasonable.

## Conclusions

CPF should be considered in the differential diagnosis when IE is suspected. Although TTE can help in the diagnosis, TEE remains the gold standard imaging modality for better characterization of valvular lesions. Surgical resection is still considered the best alternative treatment for appropriate surgical candidates. Even in asymptomatic patients, surgical resection has excellent operative and long terms outcomes. Resection can be performed safely with the preservation of the native valve. Medical treatment with anticoagulation and close monitoring could be appropriate for poor surgical candidates. The goal of intervention is to prevent embolic complications. More investigations are necessary to elucidate the precise mechanism by which CPF originates and progresses to cause the reported cardiovascular and neurovascular events.
